# F5-peptide induces aspermatogenesis by disrupting organization of actin- and microtubule-based cytoskeletons in the testis

**DOI:** 10.18632/oncotarget.11887

**Published:** 2016-09-07

**Authors:** Ying Gao, Dolores D. Mruk, Wing-yee Lui, Will M. Lee, C. Yan Cheng

**Affiliations:** ^1^ The Mary M. Wohlford Laboratory for Male Contraceptive Research, Center for Biomedical Research, Population Council, New York, USA; ^2^ School of Biological Sciences, The University of Hong Kong, Hong Kong, China

**Keywords:** testis, F5-peptide, ectoplasmic specialization, actin cytoskeleton, microtubule cytoskeleton

## Abstract

During the release of sperm at spermiation, a biologically active F5-peptide, which can disrupt the Sertoli cell tight junction (TJ) permeability barrier, is produced at the site of the degenerating apical ES (ectoplasmic specialization). This peptide coordinates the events of spermiation and blood-testis barrier (BTB) remodeling at stage VIII of the epithelial cycle, creating a local apical ES-BTB axis to coordinate cellular events across the epithelium. The mechanism(s) by which F5-peptide perturbs BTB restructuring, and its involvement in apical ES dynamics remain unknown. F5-peptide, besides perturbing BTB integrity, was shown to induce germ cell release from the epithelium following its efficient *in vivo* overexpression in the testis. Overexpression of F5-peptide caused disorganization of actin- and microtubule (MT)-based cytoskeletons, mediated by altering the spatiotemporal expression of actin binding/regulatory proteins in the seminiferous epithelium. F5-peptide perturbed the ability of actin microfilaments and/or MTs from converting between their bundled and unbundled/defragmented configuration, thereby perturbing adhesion between spermatids and Sertoli cells. Since apical ES and basal ES/BTB are interconnected through the underlying cytoskeletal networks, this thus provides an efficient and novel mechanism to coordinate different cellular events across the epithelium during spermatogenesis through changes in the organization of actin microfilaments and MTs. These findings also illustrate the potential of F5-peptide being a male contraceptive peptide for men.

## INTRODUCTION

Earlier studies have shown that the events of spermiation and BTB (blood-testis barrier) remodeling that take place at the opposite ends of the seminiferous epithelium at stage VIII of the epithelial cycle are coordinated through an autocrine-based local axis known as the apical ES-BTB-basement membrane axis (for reviews, see [1, 2]). In brief, biologically active fragments of laminin chains generated at the apical ectoplasmic specialization (apical ES, a testis-specific actin-rich anchoring junction at the Sertoli-spermatid interface) at spermiation through the action of MMP-2 (matrix metalloprotease 2) serves as the autocrine factor to induce BTB remodeling near the basement membrane [3]. Furthermore, biologically active fragments, such as NC1 (non-collagenous 1) domain, generated through the action of MMP-9 at the basement membrane, also induce BTB remodeling [4]. As such, cellular events that take place across the seminiferous epithelium throughout the epithelial cycle of spermatogenesis can be coordinated through this local autocrine-based functional and/or signaling axis. Subsequent studies have identified the active domain of the laminin-γ3 chain known as F5-peptide which has potent activity to induce reversible BTB restructuring in studies both *in vitro* and *in vivo* [5]. Furthermore, a synthetic peptide based on F5-peptide was shown to penetrate through the Sertoli cell BTB via the drug transporter Slc15a1 [6]. This observation is important since F5-peptide is generated endogenously through cleavage of the laminin-γ3 chain, a spermatid-specific protein at the apical ES in rodent testes [7–9] located outside the Sertoli cells, and it is necessary to be transported into the Sertoli cell to exert its biological activity. This finding supports the notion that F5-peptide reversibly perturbs BTB integrity via an outside-in-signaling as demonstrated earlier using different cDNA constructs [5]. In this context, it is of interest to note that recent studies using a phthalate toxicant model have also confirmed this functional axis in the testis in which phthalate-mediated MMP-2 activation was found to cleave laminin-γ3 chain at the apical ES to generate biologically active peptides, which in turn perturbed the BTB integrity near the basement membrane in the rat testis [10, 11].

Based on these earlier studies, this F5-peptide is an endogenously generated peptide having the potent activity of disrupting the BTB reversibly, and these changes were also associated with germ cell exfoliation [5]. It appears to be a novel peptide-based reversible male contraceptive. If its mechanism of action can be better understood, resources will be committed to develop this peptide into a potential male contraceptive. Herein, we report findings illustrate the molecular mechanism by which this F5-peptide induces Sertoli cell BTB restructuring *in vitro* and *in vivo*. We also provide evidence to demonstrate that the locally produced F5-peptide at the Sertoli-spermatid interface causes apical ES degeneration, leading to rapid spermatid loss from the epithelium via spermiation, besides its potent biological activity to perturb Sertoli cell TJ (tight junction)-barrier function in an *in vivo* BTB functional assay. These findings also illustrate a novel approach of male contraceptive development by perturbing the Sertoli cell cytoskeletal function.

## RESULTS

### F5-peptide perturbs ES function by disrupting distribution of BTB-associated proteins

**Figure 1:**
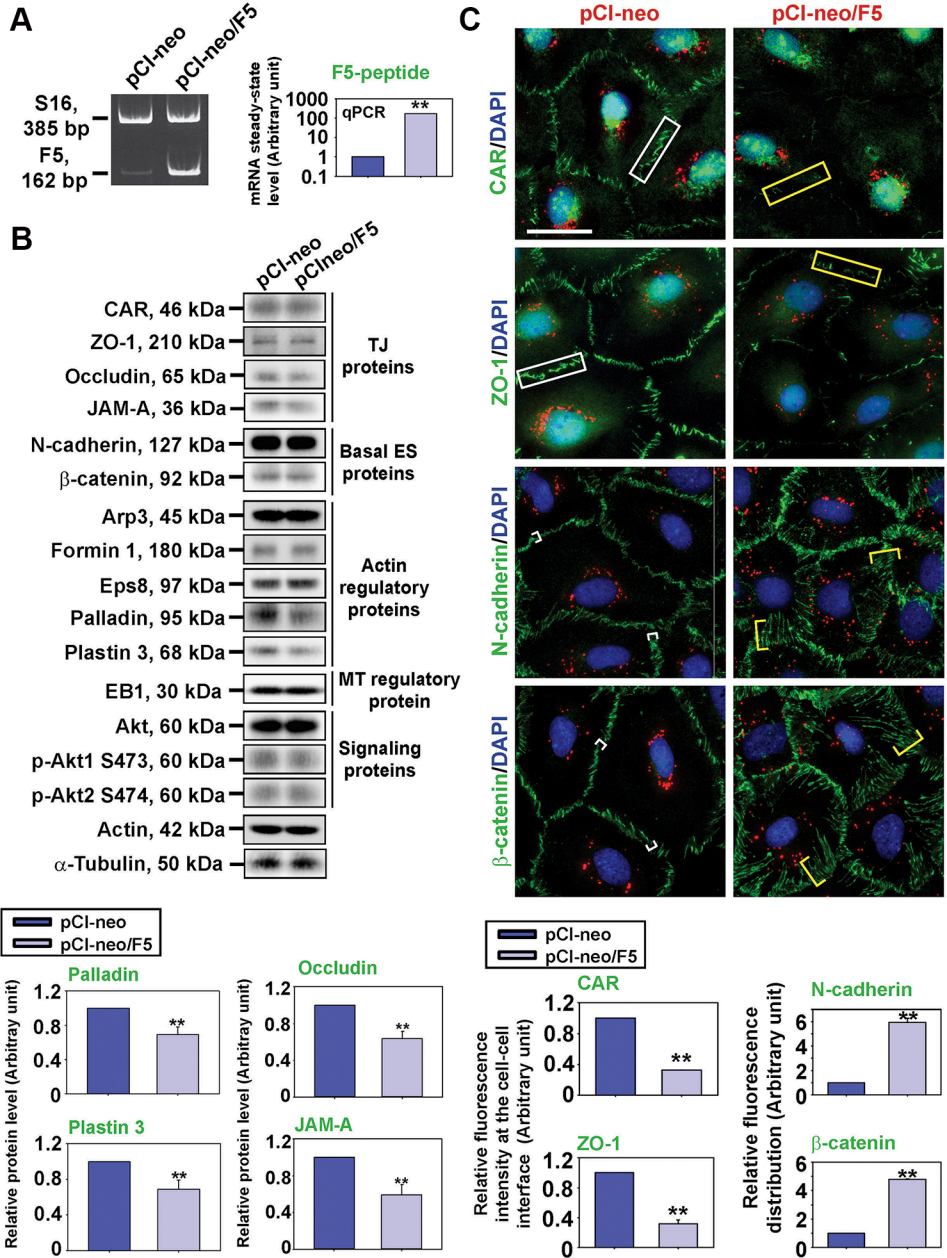
F5-peptide perturbs distribution of BTB-associated proteins at the Sertoli cell-cell interface Sertoli cells were cultured alone for 3 day, and transfected with pCI-neo/F5 *vs.* pCI-neo vector alone (control) for 24 hr. Thereafter, cells were rinsed with F12/DMEM and terminated 24 hr later for RT-PCR and IF on day 5 *vs.* 48 hr later for IB on day 6. (**A**) Successful overexpression of F5-peptide was confirmed by RT-PCR and q-PCR by using a specific primer pair (Table S2) specific to F5-peptide. S16 served as a loading control for RT-PCR. GAPDH served as an internal control for q-PCR. (**B**) The steady-state levels of TJ proteins, basal ES proteins, actin regulatory proteins, MT regulatory protein, and signaling proteins found at the BTB were analyzed by IB. Overexpression of F5-peptide caused down-regulation of actin bundling proteins palladin and plastin 3 and also TJ proteins occludin and JAM-A. Actin served as a loading control. Histograms on the lower panel illustrate the down-regulation of occludin, JAM-A, palladin and plastin 3 following overexpression of F5-peptide. Each bar is a mean ± SD of *n* = 5 experiments, and data were normalized against actin. **P < 0.01. (**C**) Localization of TJ proteins (green fluorescence) CAR and ZO-1; and basal ES proteins N-cadherin and β-catenin; were analyzed by IF. Overexpression of F5-peptide caused a considerable reduction of CAR and ZO-1 fluorescence (see white (Ctrl, pCI-neo) vs. yellow (pCI-neo/F5) rectangle boxes) at cell-cell interface, whereas N-cadherin and β-catenin were shown to be diffusely localized at the cell cortical zone (see white (Ctrl, pCI-neo) *vs.* yellow (pCI-neo/F5) brackets) but internalized. Sertoli cell nuclei were visualized by DAPI (blue). The red fluorescence of Cy3-labeled plasmid DNA confirmed the successful transfection. Histograms on the lower panel summarize the findings on the upper panel to illustrate the loss of CAR/ZO-1 *vs.* re-distribution of N-cadherin/β-catenin at or from the cell-cell interface, respectively. Each bar is a mean ± SD of *n* = 3 experiments. **P < 0.01. Scale bar, 30 μm, which applies to all micrographs.

In the testis, the adhesive function of Sertoli cells and spermatids throughout the epithelial cycle of spermatogenesis is maintained by ES (for reviews, see [12, 13]). ES is an actin-rich anchoring junction typified by the presence of actin microfilaments found in the Sertoli cell and sandwiched in-between the cisternae of endoplasmic reticulum and the apposing Sertoli-Sertoli (basal ES) or Sertoli-step 8–19 spermatid (apical ES) (for reviews, see [1, 12]). We thus examined if F5-peptide-induced TJ-permeability barrier disruption was mediated through the disorganization of actin microfilaments in Sertoli cells. Sertoli cells cultured *in vitro* for 3 days with an established functional TJ-barrier that mimicked the BTB *in vivo* was used in this study. F5-peptide, a 189 bp cDNA encoding a 63-amino acid peptide corresponding to domain IV of the laminin-γ3 chain, formerly shown to perturb the Sertoli cell TJ-barrier function *in vitro* and *in vivo* [3, 5] was cloned into the pCI-neo mammalian expression vector. Overexpression of F5-peptide (pCI-neo/F5) *vs.* empty vector (pCI-neo, control) in Sertoli cells (Figure 1A) down-regulated the expression of TJ proteins occludin and JAM-A, consistent with earlier findings [3], also actin bundling proteins palladin and plastin-3, but not others, at the BTB (Figure 1B). Furthermore, its overexpression perturbed the distribution and/or localization of TJ (e.g., CAR/ZO-1) and basal ES (e.g., N-cadherin/β-catenin) protein at the Sertoli cell-cell interface. CAR and ZO-1 were likely internalized from near the cell surface into the cell cytosol, whereas N-cadherin and β-catenin were diffusely localized at the cell-cell interface (Figure 1C) when the relative distribution of fluorescence for these two proteins were quantified (see micrographs in Figure 1C *vs.* histograms below). However, it appeared that there was a reduction in the expression of CAR and ZO-1 when the fluorescence intensity at the cell-cell interface was analyzed but not detected by immunoblotting (Figure 1C *vs.* 1B), we offered the following explanation. From multiple experiments by IB, we did not detect any changes in the expression of CAR nor ZO-1 as shown in Figure 1B, yet their fluorescence at the cell-cell interface diminished after overexpression of F5-peptide as noted in Figure 1C and summarized in the histograms below. This is likely due to the fact that these two proteins were no longer aligned exclusively at the cell cortical zone as found in control (transfected with pCI-neo alone) cells, but uniformly dispersed inside the cell cytosol (but not yet degraded via the lysosomal or ubiquitin pathway). Since they no longer formed aggregates – either at the cell-cell interface or in specific domains inside the Sertoli, similar to N-cadherin and ß-catenin, they were not notably detected, but the total steady-state levels of these two proteins namely CAR and ZO-1 remained relatively unaltered when proteins in the whole cell lysates were used for analysis by immunoblotting as shown in Figure 1B.

### F5-peptide perturbs ES function by disrupting actin microfilament organization

**Figure 2:**
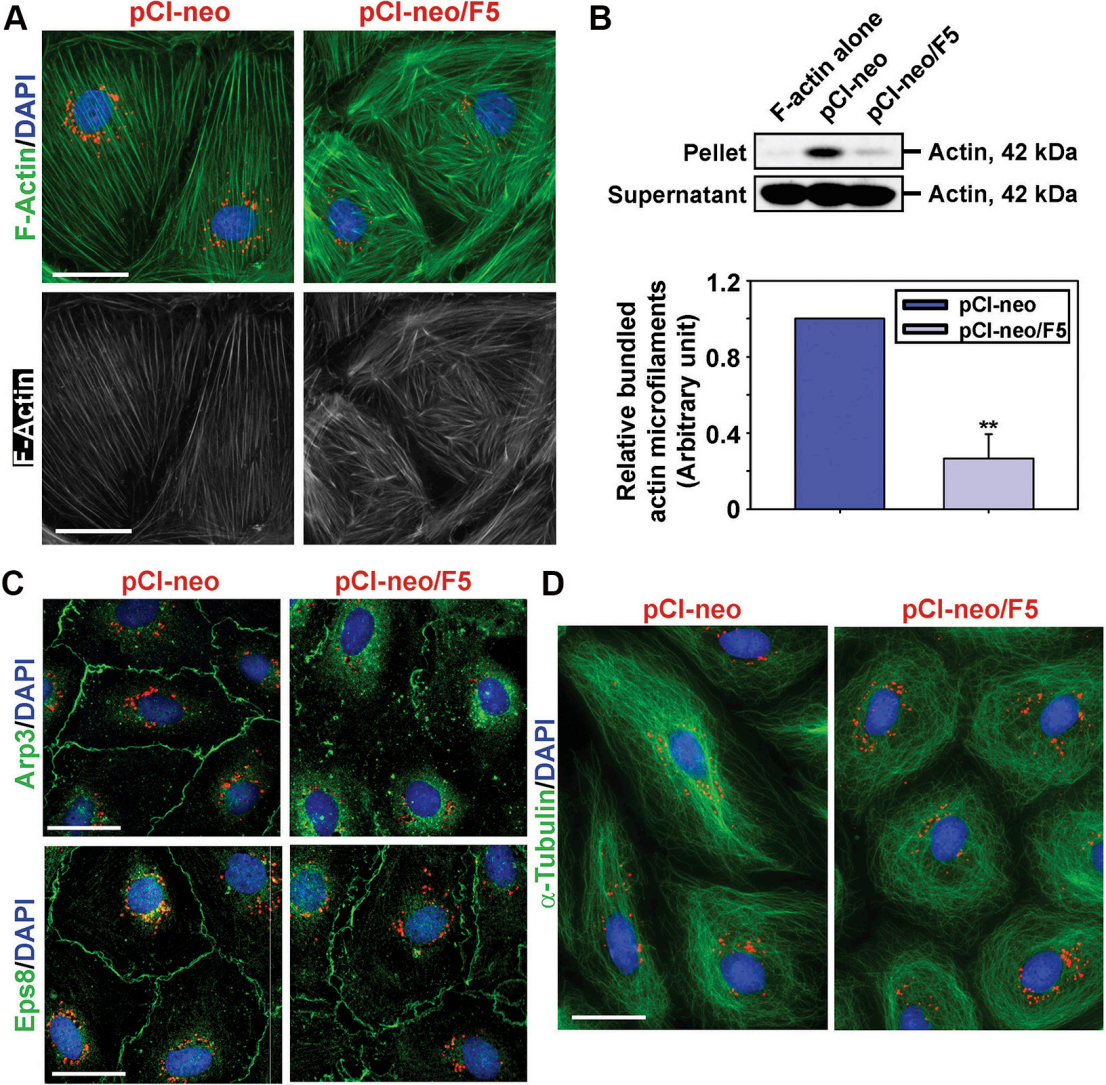
Overexpression of F5-peptide induces re-organization of actin- and microtubule (MT)-based cytoskeletons in Sertoli cells (**A**) Overexpression of F5-peptide induced disorganization of actin microfilaments in which truncation of F-actin network (green or gray) in Sertoli cells was noted. Scale bar, 30 μm, which applies to other micrographs. (**B**) Actin bundling assay was performed to illustrate overexpression of F5-peptide led to a considerable loss of actin bundling capability. In this biochemical assay, pellet contained bundled actin microfilaments, whereas supernatant contained unbundled, linear and/or truncated actin microfilaments. Histograms summarize the findings on the upper panel, and each bar is a mean ± SD of *n* = 4 experiments. **P < 0.01. (**C**) Overexpression of F5-peptide caused internalization of branched actin polymerization protein Arp3. Moreover, actin barbed end capping and bundling protein Eps8 was diffusely localized at the cell-cell interface. These changes thus led to the generation of branched and truncated actin microfilaments in Sertoli cell cytosol as noted in (A). Scale bar, 30 μm. (**D**) F5-peptide also impaired microtubule (MT) organization when MT was visualized by α-tubulin staining which is the building block of MTs. Following overexpression of F5-peptide, α-tubulin-based MTs no longer stretched across the entire Sertoli cells as noted in control cells, instead, MTs were found to round up, encircling the Sertoli cell nuclei. Scale bar, 30 μm. Sertoli cell nuclei were visualized by DAPI (blue). In A, C and D, Cy3-labeled plasmid DNAs (red) illustrate successful transfection.

Since these adhesion protein complexes all utilized F-actin for attachment, we next examined any changes in the organization of actin microfilaments in these Sertoli cells. Overexpression of F5-peptide indeed perturbed actin microfilament organization across the cell cytosol. They were found to be grossly truncated instead of laying across the cell cytosol as actin filament bundles found in pCI-neo control cells (Figure 2A). This phenotype of actin filament defragmentation following F5-peptide overexpression shown in Figure 2A was supported by findings of a biochemical assay that evaluated the relative actin bundling activity quantitatively. Interestingly, F5-peptide-caused considerable reduction of bundled actin (Figure 2B), likely the result of a re-distribution of branched actin polymerization protein Arp3. For instance, Arp3 was found to be localized considerably into the cell cytosol instead of at or near the cell surface (Figure 2C). Moreover, distribution of the actin barbed end capping and bundling protein Eps8 was also found to be diffusely localized at the cell-cell interface (Figure 2C). Thus, actin microfilaments failed to be organized to support proper localization of TJ- and basal ES-proteins near the cell surface as noted in Figure 1C. Furthermore, the organization of α-tubulin, the building block of microtubule (MT) that serves as the track for spermatid transport, was also shown to be perturbed following F5-peptide overexpression. MTs no longer stretched across the Sertoli cell cytosol but retracted by surrounding the cell nuclei instead (Figure 2D).

### F5-peptide causes defects in germ cell adhesion, and spermatid/phagosome transport

**Figure 3:**
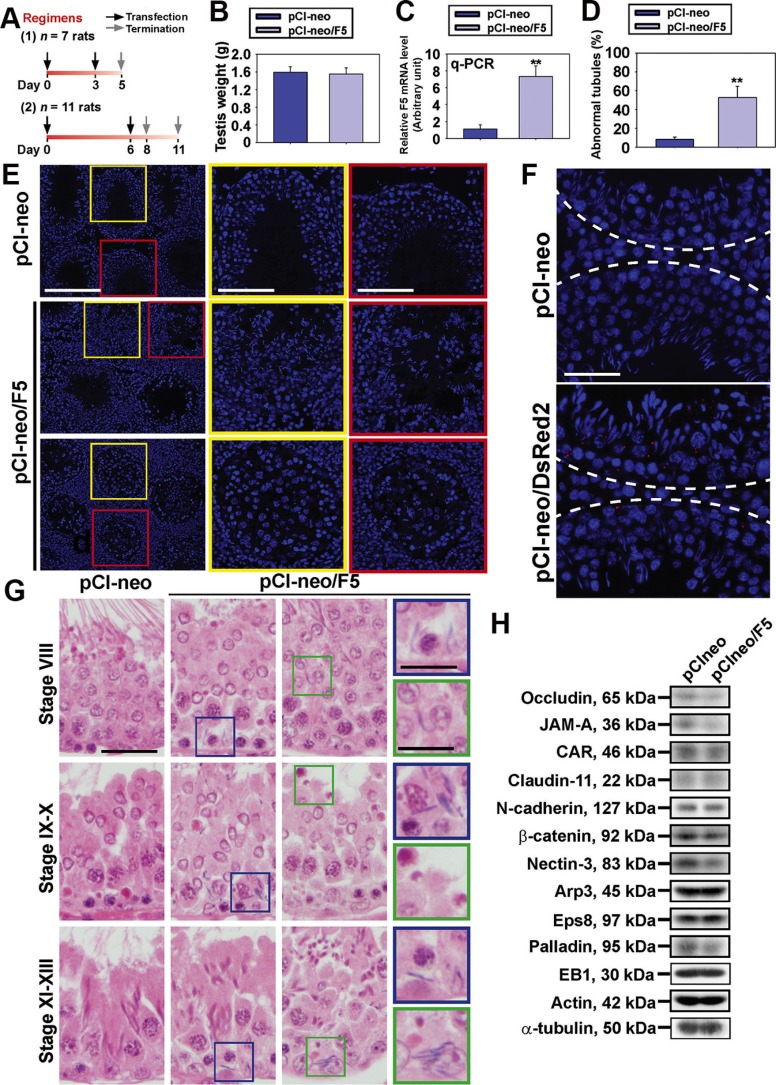
Overexpression of F5 peptide impairs spermatogenesis *in vivo* (**A**) Two regimens were used since phenotypes on day 5 (Regimen 1) vs. day 8 and 11 (Regimen 2) were similar, data were pooled for analysis. (**B**) Testis weight in control (pCI-neo) *vs*. pCI-neo/F5 in both regimens of *n* = 18 rats. (**C**) F5-peptide mRNA level was analyzed by q-PCR. GAPDH served as an internal control. Each bar is a mean ± SD of *n* = 9 rats. **P < 0.01. (**D**) Percentage of damaged tubule was assessed using frozen sections of testes (see *Materials and Methods*). Each bar is a mean ± SD of *n* = 4 rats. **P < 0.01. (**E**) Morphological analysis using frozen sections stained with DAPI. Boxed areas in yellow or red were magnified and shown in insets. Germ cell loss was detected in tubules within 48 hr following the last transfection to overexpress F5-peptide. Scale bar in first column, 150 μm; 70 μm in yellow and red boxed micrographs. (**F**) Transfection efficiency was assessed by transfecting rat testes (*n* = 3 rats) with pCI-neo vs. pCI-neo/DsRed2 using Regimen 1. Positive transfection was confirmed by > 10 DsRed2 red fluorescence aggregates in the epithelium. Scale bar, 30 μm. (**G**) Histological analysis using paraffin sections of testes. In control testes transfected with pCI-neo empty vector, elongated spermatids line-up near the tubule lumen edge in stage VIII tubules to prepare for spermiation, but elongated spermatids remain entrapped inside the epithelium in stage VIII tubules after F5-peptide overexpression. At stages IX-X and XI-XIII tubules in control testes, no step 19 elongated spermatids were found in the epithelium, and phagosomes were transported to the base of epithelium. However, similar staged tubules following F5-peptide overexpression led to retention of elongated spermatids embedded deep inside the epithelium, and phagosomes were found near the luminal edge (see blue or green boxed area). Scale bar in the first column, 30 μm, which applies to 2nd/3rd column; scale bar in the last column, 10 μm. (**H**) Steady-state levels of the TJ-, basal/apical ES-, and actin or MT regulatory proteins were analyzed by IB with actin and α-tubulin served as loading controls. These are representative findings of *n* = 9 rats.

Earlier studies showed that F5-peptide or domain IV of the laminin-γ3 chain was capable of causing BTB disruption *in vitro* and *in vivo* [3, 5]. However, it was not known if germ cell exfoliation seen in 4-week after intratesticular injection of the synthetic F5-peptide was the result of the BTB disruption or a direct effect of the F5-peptide. To assess if F5-peptide that is generated at spermiation can potentiate apical ES disruption to facilitate sperm release, we examined if there were defects in germ cell adhesion shortly following overexpression of F5-peptide *vs.* pCI-neo vector (control). Testes of adult Sprague-Dawley rats (~270-300 g b.w.) were transfected with F5-peptide using Polyplus *in vivo*-jetPEI transfection reagent at a ~55% efficiency. In both two regimens when testes were transfected twice with pCI-neo/F5 and terminated shortly thereafter (Figure 3A), there were no significant changes in testis weight between the two groups, pCI-neo/F5 *vs.* pCI-neo (Figure 3B) since depleted germ cells were in the epididymis on day 11. Their body weight also did not change noticeably between the control (pCI-neo) and pCI-neo/F5 group at 312 ± 14 and 308 ± 18 (*n* = 10 rats) on day 11, respectively. q-PCR results also confirmed overexpression of F5-peptide in the testis in both Regimens (Figure 3C). Interestingly, germ cell loss as manifested by the presence of germ cells in tubule lumen was notably detected that occurred within 2 days in Regimen 1 (i.e., day 5) and 2 (i.e., day 8) (Figure 3D). As noted in representative findings shown in Figure 3E using frozen sections of testes following DAPI staining, > 50% of the tubules had obvious signs of abnormality. These abnormal tubules were typified by the presence of germ cells in tubule lumen and extensive loss of elongating/elongated spermatids from the epithelium within 2 days following completion of transfections in Regimens 1 and 2, to be followed by the loss of round spermatids and spermatocytes 3 days thereafter in Regimen 2 (i.e., day 11) (Figure 3E). The transfection efficacy was also estimated by transfecting testes with a *Discosoma sp.* red fluorescence protein DsRed2 cloned into the pCI-neo using Regimen 1, and fluorescence aggregates of pCI-neo/DsRed2 was randomly scored in ~100 tubules from a rat testis with *n* = 3 rats on day 5 (Regimen 1) (Figure 3F). Successful transfection referred to the cross-section of a seminiferous tubule having at least 10 aggregates of red fluorescence. At least 55% of the scored tubules were found to be positive using PolyPlus *in vivo*-jetPEI as a transfection medium (Figure 3F) *vs.* ~25–30% using other conventional transfection medium as earlier reported [14, 15], illustrating a considerable increase in transfection efficiency. It is noted that the > 50% of abnormal tubules shown in Figure 3D–3E did not include tubules with defects in spermatid or phagosome transport which were better detected in histological analysis using paraffin embedded sections following H&E staining as shown in Figure 3G. In short, gross defects in the transport of spermatids and phagosomes in F5-peptide overexpressed testes were detected in the epithelium. For instance, stage 19 spermatids in stage VIII tubules were found near the base or in the center of the seminiferous epithelium when they should have been transported near the tubule lumen (Figure 3G, top panel). These step 19 spermatids persistently found in stage IX-X tubules when step 8 and 9 spermatids were detected near the base of the epithelium, and phagosomes remained near the tubule lumen when they should have been transported to the base of the epithelium for degradation (for reviews, see [16, 17]). (Figure 3G, middle panel). Furthermore, these step 19 spermatids were even detected near the base of the tubule in stage XI-XIII tubules (Figure 3G, bottom panel), illustrating gross defects in spermatid and phagosome transport following overexpression of F5-peptide. Findings by immunoblotting illustrated F5-peptide caused a down-regulation on the expression of occludin and JAM-A in the testis (Figure 3H), consistent with earlier findings [3, 5] and also *in vitro* findings (see Figure 1B). A down-regulation of an apical ES and spermatid-specific adhesion protein nectin-3 and actin bundling protein palladin in the testis following F5-peptide overexpression were also detected (Figure 3H).

### F5-peptide perturbs BTB mediated by alterations in TJ/basal ES protein distribution

**Figure 4:**
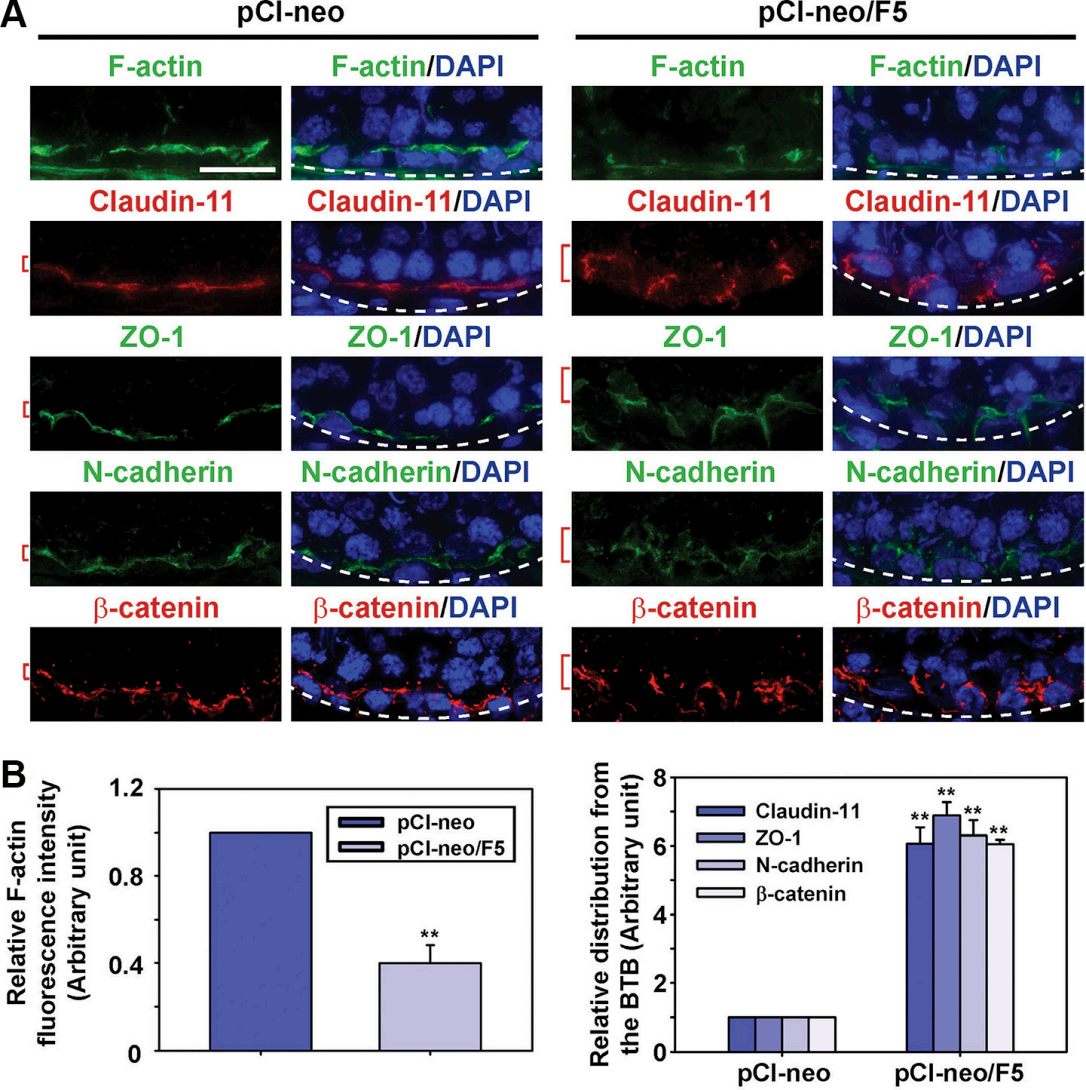
Overexpression of F5-peptide perturbs distribution TJ and basal ES proteins at the BTB through changes in the organization of F-actin in adult rat testes *in vivo* (**A**) Frozen sections obtained from testes transfected with pCI-neo/F5 vs. pCI-neo alone (control) were stained for F-actin, TJ proteins claudin-11 and ZO-1, as well as basal ES proteins N-cadherin and β-catenin. Cell nuclei were visualized by DAPI (blue). The fluorescence intensity of F-actin at the BTB was considerably diminished after F5-peptide overexpression, consistent with *in vitro* observation due to truncation and dis-organization of actin microfilaments at the site. TJ proteins claudin-11 and ZO-1, as well as basal ES proteins N-cadherin and β-catenin were found to be diffusely localized at the Sertoli cell BTB in the testis transfected with pCI-neo/F5, supporting findings *in vitro* that illustrate an increase in protein internalization at the site. Basement membrane was annotated by a dashed white line illustrating the relative location of the basement membrane/tunica propria. Scale bar, 30 μm, which applies to all other micrographs. (**B**) Semi-quantitative analysis of fluorescence data shown in (A) including fluorescence intensity (left panel) *vs.* mis-localization of TJ and basal ES proteins by diffusing away from the BTB (right panel). Data in the control group were arbitrarily set at 1 against which statistical comparison was performed. Each bar is a mean ± SD of *n* = 4 rats. **P < 0.01.

We next examined changes in the distribution of F-actin *vs.* adhesion protein complexes at the TJ (e.g., claudin-11/ZO-1) and the basal ES (e.g., N-cadherin/β-catenin) following overexpression F5-peptide and compared to control testes transfected with pCI-neo vector alone (control). It was noted that overexpression of F5-peptide reduced the expression of F-actin at the BTB (Figure 4A, 4B). Due to the F5-peptide-induced loss of F-actin which also served as the attachment site for TJ and basal ES adhesion protein complexes, TJ proteins claudin-11 and ZO-1, as well as basal ES proteins N-cadherin and β-catenin no longer tightly associated with the BTB, instead, these proteins were diffusely localized at the BTB (Figure 4A, 4B). These findings suggest that the BTB integrity *in vivo* following overexpression of F5-peptide might have been compromised.

### Overexpression of F5-peptide in the testis induces BTB disruption

**Figure 5:**
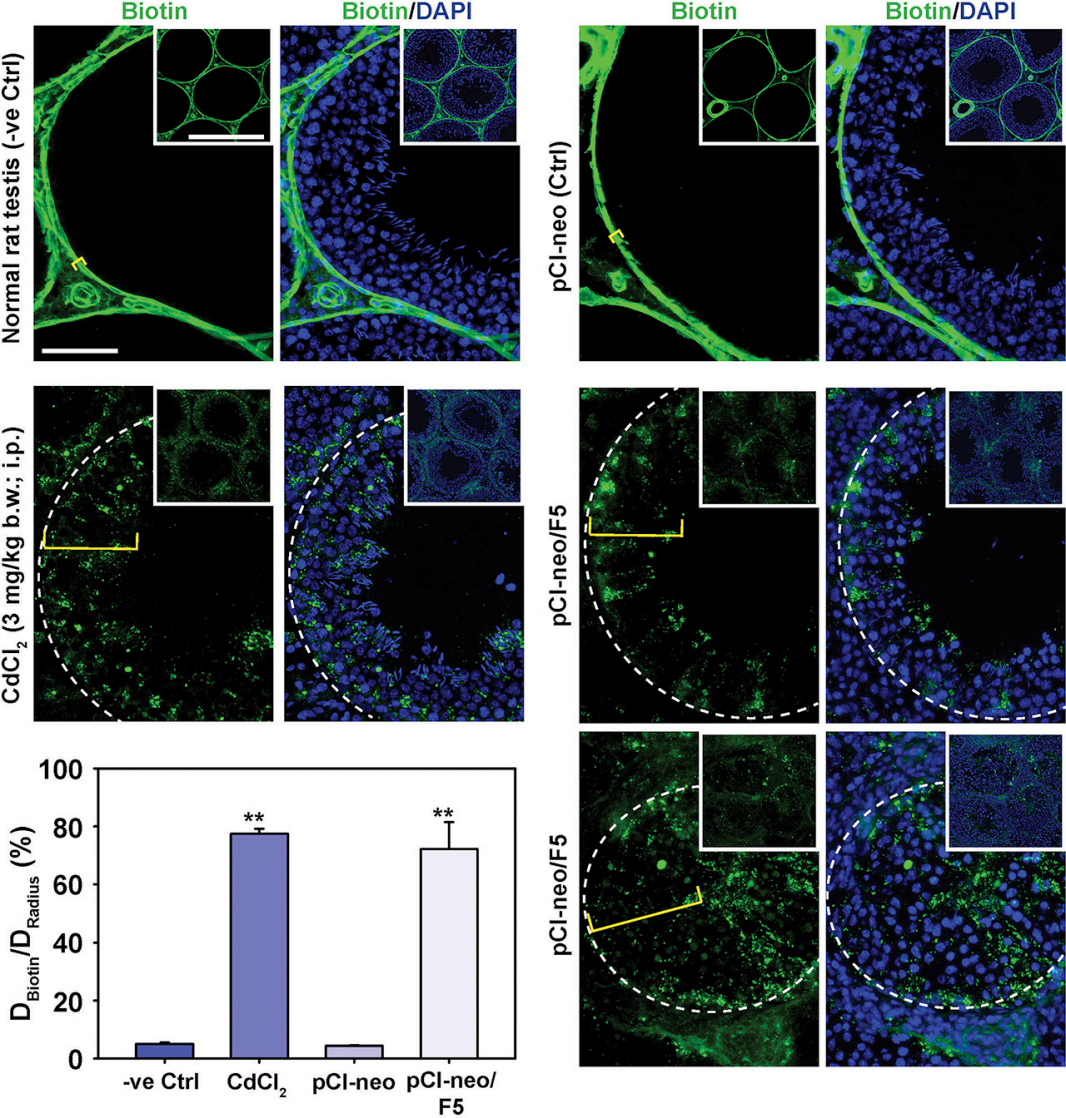
Overexpression of F5-peptide in the test is perturbs BTB function *in vivo* BTB integrity assay was performed to assess the ability of an intact BTB to block the diffusion of a small molecule biotin (EZ-Link Sulfo-NHS-LC-Biotin, Mr 556.59) across the BTB which biotinylated proteins in the adluminal compartment. In normal rat testes and testes transfected with pCI-neo alone (control), the functional BTB blocked biotin reagent from entering the adluminal compartment so that only proteins at the BTB and the interstitial space were labeled. Biotinylated proteins were subsequently visualized using frozen sections of the testis and stained with Alexa Fluor 488-streptavidin (green fluorescence). Distance traveled by biotin beyond the basement membrane/tunica propria was annotated by a yellow bracket. In control testes, biotin failed to travel beyond the basement membrane/tunica propria which was annotated by a dashed white line. In testes from rats treated with CdCl_2_ (positive control) or transfected with pCI-neo/F5, biotin penetrated well into the adluminal compartment, illustrating that F5-peptide perturbed the BTB integrity. Lower magnified images of ~3–4 tubules were shown in insets and the enlarged image of a typical tubule was shown in the micrograph. Scale bars, 50 μm, and 200 μm in insets, which applies to corresponding micrographs and/or insets. Histograms show the semi-quantitative data by comparing the distance of biotin traveled into the epithelium (D_Biotin_) *vs.* the radius of seminiferous tubule (D_Radius_). For oblique sections, the radius of the tubule was obtained by averaging the longest and shortest distance from the basement membrane. Each bar is a mean ± SD of ~50 tubules that were randomly selected and scored from testes of 6 rats with a total of 300 randomly selected tubules. **P < 0.01.

Using an *in vivo* functional BTB integrity assay, overexpression of F5-peptide in the testis effectively perturbed BTB integrity within 2 days following two consecutive transfection with pCI-neo/F5, making the tubules freely accessible by biotin (Figure 5). This phenotype was similar to treatment of testes with CdCl_2_, which is known to perturb BTB integrity effectively *in vivo* [18, 19]. It is also noted that biotin was detected in all the tubules in F5-peptide overexpressed testes, even in tubules appeared to have normal spermatogenesis by DAPI staining, the BTB integrity was perturbed. Interestingly, these findings thus illustrate that the transfection efficiency was in fact higher than 55% as we had considered a tubule to be positively transfected when at least 10 aggregates of DsRed2 were detected across the epithelium (see Figure 3F). These findings illustrate that F5-peptide is an effective agent to perturb BTB integrity, likely through its disruptive effects on actin organization at the BTB (Figure 4).

### F5-peptide effectively perturbs apical ES function

**Figure 6:**
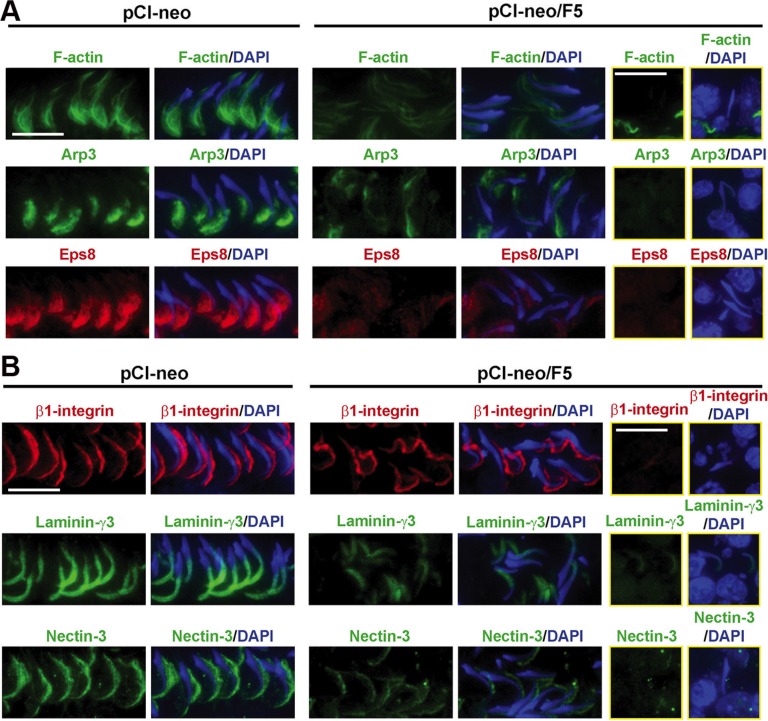
Overexpression of F5-peptide perturbs F-actin organization through changes in the spatial expression of actin regulatory proteins, which in turn disrupts apical ES protein distribution in adult rat testes *in vivo* Frozen sections of testes following transfection of pCI-neo/F5 *vs.* pCI-neo (vector alone, control) were stained for F-actin, Arp3, and Eps8; as well as integral membrane proteins at the apical ES: β1-integrin (Sertoli cell-specific) *vs.* spermatid-specific laminin-γ3 chain and nectin-3. Spermatids that were entrapped inside the seminiferous epithelium were shown in the two rectangular columns on the right. Cell nuclei were visualized by DAPI. (**A**) At stage VII, F-actin, actin regulatory proteins Arp3 and Eps8 were prominently localized to the concave side of spermatid heads in control testes. Overexpression of F5-peptide, however, induced mis-organization of F-actin which was considerably diminished at the apical ES found in elongated spermatids located near the tubule lumen or embedded inside the epithelium. These changes in F-actin organization were the result of changes in spatial expression of Arp3 and Eps8 since these proteins no longer restricted to the concave side of spermatids, and their expression was considerably diminished. Furthermore, many spermatids had lost their polarity since they no longer pointed toward the basement membrane as found in control tubules. For spermatids entrapped inside the epithelium in these tubules, the fluorescence signals of Arp3 and Eps8 were also considerably diminished after F5-peptide overexpression, illustrating apical ES in these spermatids was also disrupted. Scale bar, 10 μm, which applies to other micrographs. (**B**) Apical ES adhesion proteins β1-integrin and nectin-3 were localized at the convex side of spermatid heads whereas laminin-γ3 chain was at the tip of spermatid heads in normal testes. Following F5-peptide overexpression, β1-integrin was grossly mis-localized since some fluorescence signal was found on the concave side of spermatid heads; whereas laminin-γ3 chain and nectin-3 were also considerably down-regulated and mis-localized, thereby impeding spermatid adhesion, leading to germ cell exfoliation as noted in Figure 3. Scale bar, 10 μm, which applies to other micrographs in the same panel.

We next examined if F5-peptide also disrupts actin organization at the apical ES to induce spermatid exfoliation. Overexpression of F5-peptide in the testis indeed altered the spatiotemporal expression of F-actin, possibly through changes in the spatial expression of Arp3 and Eps8. These two actin regulatory proteins known to confer actin microfilaments to their bundled configuration at the apical ES were grossly affected *vs.* control testes transfected with pCI-neo empty vector (Figure 6A). These changes thus caused apical ES degeneration, facilitating the release of spermatids analogous to spermiation as noted in Figure 3D, 3E. More important, these changes associated with apical ES disruption were not limited to spermatids located near the tubule lumens in these late stage VII tubules following overexpression of F5-peptide (pCI-neo/F5) (two middle columns), but also for spermatids embedded inside the epithelium as seen in the two right panels (boxed in yellow) since these elongated spermatids were intermingled with round spermatids and/or spermatocytes (Figure 6A). The disruption of actin organization further led to disruptive localization of apical ES protein β1-integrin (Sertoli cell-specific) or down-regulation of apical ES proteins laminin-γ3 chain (spermatid-specific), and nectin-3 (spermatid-specific), both in spermatids located near the tubule lumen (two middle panels, Figure 6B) and spermatids embedded inside the epithelium (two right yellow-boxed panels – note the presence of round spermatids or spermatocytes intermingled with elongated spermatids, Figure 6B) following overexpression of F5-peptide (pCI-neo/F5) *vs.* control testes (pCI-neo vector alone) (Figure 6B). This is possibly due to the fact that apical ES proteins utilized F-actin for their attachment. In this context, it is of interest to note IF results herein showed that the expression intensity of Arp3 and Eps8 was reduced in F5-peptide overexpressed testes, unlike data shown in Figure 3H that the steady-state protein levels of Arp3 and Eps8 were not affected. It must be noted that results for Arp3 and Eps8 at the apical ES shown in Figure 6A were from stage VII tubules (stage VII tubules account for ~20% of all tubules [20]), which is also the stage wherein their fluorescence intensity was reduced after F5-peptide overexpression. Also, Arp3 and Eps8 are not restricted to the apical ES. For instance, Eps8 is highly expressed at both apical ES and basal ES/BTB from stages V-VI [21] and Arp3 is also highly expressed at the basal ES/BTB in VIII [22]. The IB results in Figure 3H, however, showed the steady-state protein levels of Arp3 and Eps8 in the whole testis which include seminiferous tubules of all stages.

### Overexpression of F5-peptide disrupts F-actin organization in the testis

**Figure 7:**
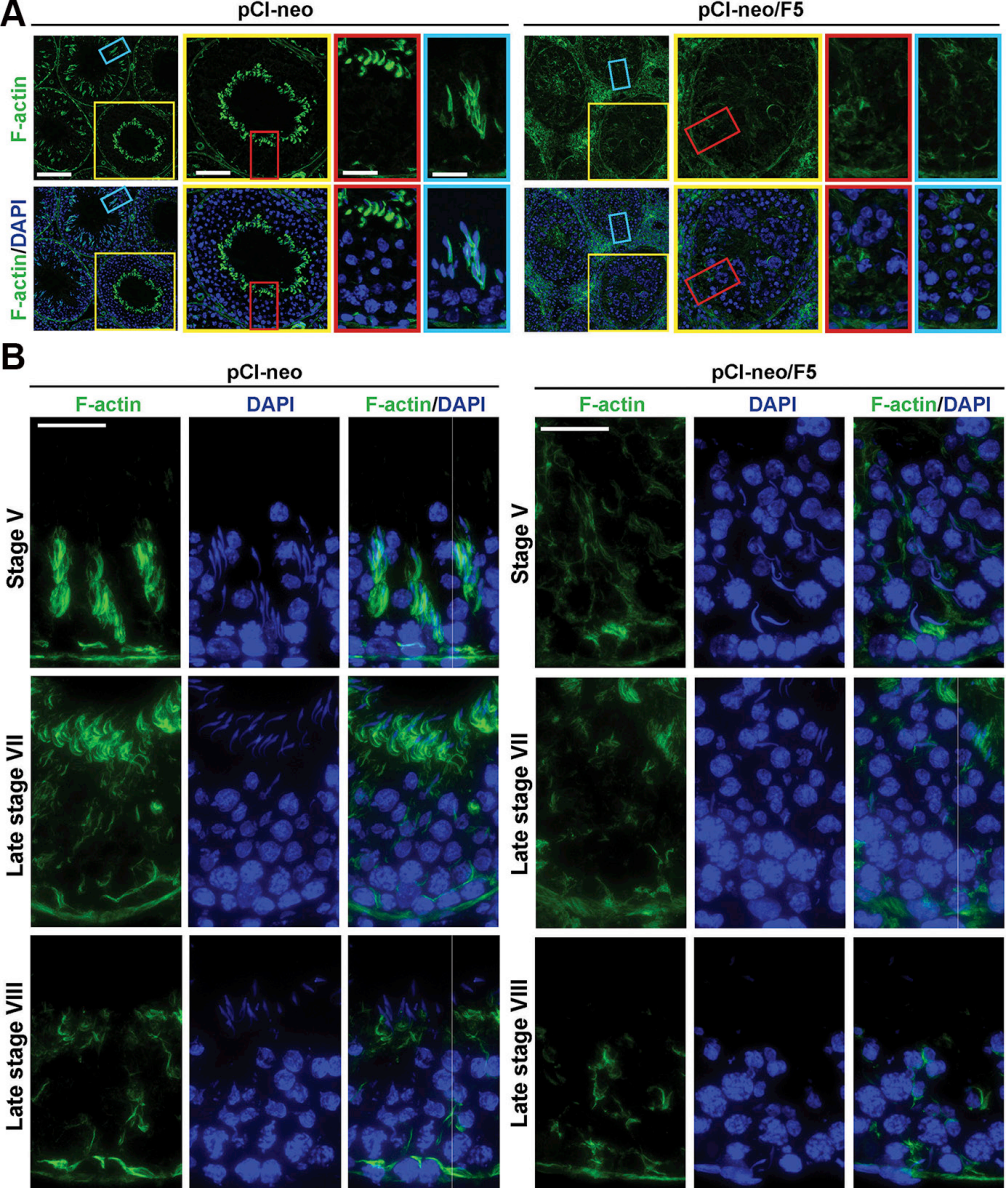
Overexpression of F5-peptide perturbs F-actin organization in the seminiferous epithelium of adult rat testes *in vivo* Rat testes were transfected with pCI-neo/F5 *vs.* pCI-neo (control) using Regimens (*n* = 9 rats) shown in Figure 3A. Frozen sections of testes were used to visualize the distribution of F-actin by FITC-phalloidin. Cell nuclei were visualized by DAPI. (**A**) Overexpression of F5-peptide led to extensive germ cell loss in tubules wherein F-actin distribution was grossly disrupted. The typical “stalk-like” structures of F-actin was not found in tubules following overexpression with F5-peptide. Scale bar in the left column, 100 μm; Scale bar in yellow box, 50 μm; red or blue box, 15 μm. (**B**) Representative micrographs of frozen sections of normal testes in selected stages were stained for F-actin. As noted herein, F-actin appeared as “stalk-like” structures, tightly associated with elongating spermatids in transport across the epithelium in stage V tubules, also associated with apical ES surrounding the head of elongated spermatids in late stage VII, but considerably diminished in late stage VIII tubules to facilitate the release of sperms at spermiation. Moreover, some stalk-like structure reappeared across the epithelium in late stage VIII, perhaps being used to support the transport of residual bodies/phagosome to the basal compartment for their eventual lysosomal degradation. However, following F5-peptide overexpression, the typical F-actin organization in the epithelium was considerably disrupted, For instance, elongating spermatids in stage V tubules no longer associated with the F-actin-based stalk-like structures and F-actin was considerably diminished in stage VII-VIII tubules. Elongated spermatids, however, remained entrapped inside the epithelium even though the apical ES had been disrupted. Scale bar, 25 μm, which applies to other micrographs.

F-actin, besides serving as the attachment site for apical and basal ES adhesion protein complexes, is also known to be involved in spermatid and intracellular organelle (e.g., phagosomes, residual bodies) transport for reviews, see [16, 23, 24]. Thus, in order to better understand the mechanism by F5-peptide induced germ cell exfoliation in pre-stage VIII tubules, and also caused entrapment of step 19 spermatids in post-stage VIII tubules, we examined changes in the gross organization of F-actin in the seminiferous epithelium following overexpression of F5-peptide. In control testes transfected with empty vector, F-actin was prominently detected at the apical ES and basal ES/BTB but also the tunica propria surrounding the base of each seminiferous tubule (Figure 7A, 7B). F-actin was also predominantly found to be associated with elongating spermatids being transported across the epithelium, such as in stage V, XII or XIII tubule, apparently serving as the track to support spermatid transport (Figure 7A, left panels). However, following overexpression of F5-peptide, F-actin was considerably down-regulated and F-actin no longer organized as distinctive track-like structures across the epithelium as seen in control testes (Figure 7A, right panels). These changes were more obvious in the magnified images shown in Figure 7B in which overexpression of F5-peptide considerably perturbed F-actin organization in the seminiferous epithelium, and the distinctive track-like structures across the epithelium were rarely found in tubules following overexpression with F5-peptide vs. control testes with distinctive track-like structures shown in stage V tubules. It is note that these track-like structures were somewhat diminished in late stage VII and late stage VIII tubules when the transport of elongated spermatids to the adluminal edge of tubule lumen was completed. It is likely that this considerably disruption of F-actin organization failed to support spermatid adhesion, leading to their premature release into the tubule lumen. Also, the absence of the actin conferred track-like structure impeded the transport of spermatids inside the epithelium even though apical ES adhesion function was disrupted, causing entrapped of late stage spermatids in the epithelium as noted in Figure 6.

### Overexpression of F5-peptide disrupts MT organization in the testis

**Figure 8:**
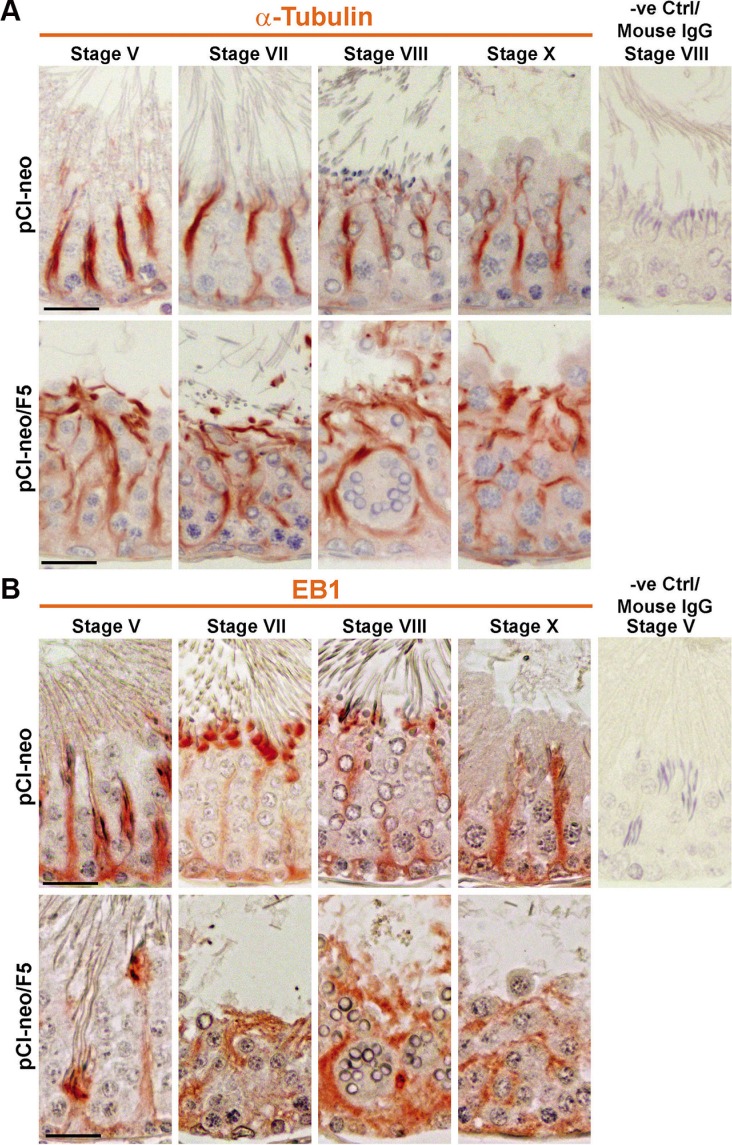
Overexpression of F5-peptide perturbs MT organization in the seminiferous epithelium of adult rat testes *in vivo* Rat testes were transfected with pCI-neo/F5 *vs.* pCI-neo empty vector (control) using regimens (*n* = 9 rats) shown in Figure 3A. Testes were used for immunohistochemistry to visualize the distribution of α-tubulin, the building block of microtubules (MTs) in the seminiferous epithelium. (**A**) In normal testes, MTs appeared as track-like structures were found longitudinally across the epithelium to provide the tracks to support the transport of organelles (e.g., phagosomes, endocytic vesicles) and also spermatids in virtually all stages of the epithelial cycle. Following overexpression of F5-peptide, on day 2 or day 5 after completion of transfection, tracks were grossly affected. They no longer distinctively found across the epithelium, but truncated and some laid across the epithelium in parallel to the tunica propria. Moreover, MTs were also found to engulf round spermatids, becoming the precursors of giant multinucleated round spermatids, destined to be degenerated. The last micrograph on the right of the top panel is the negative control (a stage VIII tubule) in which the primary antibody was substituted with the mouse IgG. Scale bar, 30 μm, which applies to other micrographs. (**B**) Changes in the organization of EB1, a +TIP protein known to bind to MTs, helping to stabilize MTs, were also noted in testes following overexpression of F5-peptide. Similar to α-tubulin, EB1 appeared as track-like structures across the epithelium, but overexpression of F5-peptide led to either considerably loss of EB1 in the epithelium, and it no longer laid across the epithelium longitudinally, but truncated and also wrapped around groups of round spermatids destined to become giant multinucleated round spermatids for eventual degeneration. The last micrograph on the right of the top panel is the negative control (a stage V tubule) in which the primary antibody was substituted with the mouse IgG. Scale bar, 30 μm, which applies to other micrographs.

It is generally accepted that MT conferred the tracks to support cellular transport in mammalian cells and tissues, such as spermatids, residual bodies and phagosomes in the testis (for reviews, see [25, 26]). We thus examined if overexpression F5-peptide perturbed the organization of MTs in the seminiferous epithelium. Since α-tubulin is the building block of MT which is composed of dimeric α−/β-tubulin (for a review, see [26]), we examined the organization of MTs in the seminiferous epithelium by staining α-tubulin in cross-sections of testes. As noted in control testes, prominent MT tracks were found that laid across the epithelium as stalks, serving as the tracks for the transport of spermatids, residual bodies and/or phagosomes in stage V, VII, VIII and X tubules (Figure 8A). Following overexpression of F5-peptide in the testis, prominent track-like MT structure were no longer detected. These longitudinally aligned tracks were truncated, and some even laid horizontally, parallel to the tunica propria, and some even rounded up to enclose round spermatids, as precursors of the giant multinucleated cells (Figure 8A). EB1 (end-binding protein 1), a plus-end tracking protein (+TIP), is known to preferentially bind to the plus (+)−end of a polarized MT to promote MT growth (for reviews, see [26, 27]), was also found to associate with the MT-based track-like structures across the epithelium in control testes (Figure 8B). However, the track-like structures stained for EB1 was considerably diminished following overexpression of F5-peptide, they were either truncated, laying horizontally and paralleled to the tunica propria and some also rounded up the round spermatids, the precursors of giant multinucleated round spermatids (Figure 8B). These findings illustrate that the tracks conferred by MTs were considerably disrupted (Figure 8), so that elongated spermatids embedded inside epithelium could not be transported to the tubule lumen to be released but were trapped instead even though their apical ES was disrupted as noted in Figure 6.

## DISCUSSION

It appears that F5-peptide can serve as a novel male contraceptive peptide that is endogenously produced since high doses of F5-peptide administered to the testis is known to reversibly perturb the BTB function that leads to massive germ cell exfoliation [5]. This finding is consistent with earlier studies using toxicant models in which permanent BTB disruption led to germ cell exfoliation and male infertility including treatment of rodents with cadmium [18, 19, 28] or glycerol [29]. Herein, we provide evidence that the F5-peptide also perturbs spermatid adhesion per se since its overexpression induces apical ES degeneration across the epithelium in all stages of the epithelial cycle. This finding is important since it illustrates the F5-peptide generated locally in the seminiferous epithelium near the tubule lumen can potentiate further break-down of apical ES to facilitate spermiation. In short, besides exerts its effects at the basal ES via intracellular signaling, it also facilitates apical ES degeneration, serving as a local autocrine peptide. Furthermore, F5-peptide also perturbs desmosome function since round spermatids and spermatocytes anchor to the Sertoli cells via desmosome (for a review, see [30]). In short, apical ES and desmosome degeneration occurs almost simultaneously along with the BTB disruption, within ~2-days following completion of the overexpression regimen as reported herein. Thus, apical ES and desmosome degeneration that leads to germ cell exfoliation apparently is not secondary to the BTB disruption. Collectively, these findings form the basis of a novel concept that there is local autocrine-based regulatory axis that regulates and coordinates cellular events at the apical and basal ES, across the seminiferous epithelium.

Here, we have shown that F5-peptide employs an efficient system to coordinate cellular events in the apical ES-BTB axis through changes in the organization of the actin- and MT-based cytoskeleton. It is noted that Sertoli cells are the only somatic cell type in the seminiferous epithelium that nurture multiple germ cells at a Sertoli:germ cell ratio of ~1:30-1:50 [31], and there is only a single layer of Sertoli cells across the entire seminiferous epithelium in which germ cells at different stages of their development are connected to the somatic Sertoli cells through different anchoring junctions including apical ES, gap junction and desmosome. As such, the sites where spermiation and BTB remodeling that take place at the opposite ends of the epithelium are indeed connected through the underlying cytoskeletal networks. Thus, cellular events that occur at the opposite ends of the epithelium can be regulated or coordinated through the actin- and MT-based cytoskeletons. As shown herein, F5-peptide efficiently perturbs actin microfilament and MT organization across the epithelium through changes in the spatiotemporal expression of the actin regulatory proteins, such as branched actin polymerization protein Arp3 and barbed-end capping and bundling protein Eps8. This, in turn, rapidly re-organizes actin microfilaments and MTs, converting them between a bundled *vs.* a branched/truncated configuration, modulating adhesion function at the apical and basal ES. Furthermore, these changes do not require any de novo synthesis of proteins, perhaps not even transcriptional modification of genes and/or post-translational modification of proteins, providing an efficient system of regulation since millions of germ cells are processed through different stages of the epithelial cycle along the length of the seminiferous tubule. Furthermore, these changes also modulate endocytic vesicle-based intracellular trafficking events, including cell signaling, based on studies in other mammalian cells and tissues [32, 34], suggesting that F5-peptide likely exerts its effects through similar mechanisms by modifying intracellular trafficking events. For instance, it is possible that changes in the endocytic vesicle-mediated protein trafficking perturb the spatial expression of Arp3 and Eps8 at the Sertoli cell-cell interface following overexpression of F5-peptide. Additionally, it is likely that some signaling proteins are also involved in the F5-peptide-mediated ES disruption. It is known that p-FAK-Tyr^407^ is involved in BTB remodeling through its effects on the N-WASP-Arp3-induced actin microfilament organization at the basal ES in Sertoli cells [35]. On the other and, p-FAK-Tyr^397^ is also involved in actin microfilament organization at the apical ES in the rat testis [14]. In fact, overexpression of a constitutively active p-FAK-Tyr^407^ mutant of p-FAK-Y407E can block the F5-peptide-induced Sertoli cell TJ-permeability barrier function [5]. Taken collectively, these data illustrate the likely involvement of FAK, perhaps other protein kinases, in F5-peptide-mediated ES dynamics. This possibility must be carefully evaluated in future studies.

In summary, we have demonstrated unequivocally herein that F5-peptide induces apical and basal ES restructuring in the rat testis through its effects on the underlying F-actin-based and MT-based cytoskeletons by altering the spatial expression of the actin- and MT-binding and regulatory proteins.

## MATERIALS AND METHODS

### Animals and antibodies

Male Sprague-Dawley rats, pups and adults (~270~300 g b.w.), were purchased from Charles River Laboratories (Kingston, NY). The use of rats for experiments reported herein was approved by the Rockefeller University Institutional Animal Care and Use Committee (IACUC) with Protocol Numbers 12-506 and 15-780-H. All methods and experimental protocols used for relevant studies reported herein were approved by and carried out in accordance with relevant guidelines and regulations of the Rockefeller University Laboratory Safety and Environmental Health, the Rockefeller University Institutional Biosafety Committee (IBC), and the Rockefeller University Comparative Bioscience Center (CBC). These methods were also described in detail in the sections below. Rats were euthanized by CO_2_ asphyxiation using slow, at 20%–30%/min, displacement of chamber air with compressed CO_2_ in a chamber with a built-in regulator approved by the Rockefeller University Laboratory Safety and Environmental Health. Antibodies were obtained commercially, unless specified otherwise, and listed in Table S1.

### Primary Sertoli cell cultures

Sertoli cells were isolated from the testes of 20-day-old rats as described [36]. Freshly isolated Sertoli cells were seeded on Matrigel (1:7, diluted in F12/DMEM; BD Biosciences, San Jose, CA)-coated culture plates (for lysate preparation, RNA isolation and actin bundling assay) or coverslips (for immunofluorescence (IF) microscopy) at densities 0.5 and 0.04 × 10^6^ cells/cm^2^, respectively. Sertoli cells were cultured in serum-free F12/DMEM (Life Technologies, Carlsbad, CA) supplemented with growth factors and gentamicin as described [36] in a humidified atmosphere of 95% air/5% CO_2_ (v/v) at 35°C. On day 2, ~30 hr following their isolation, Sertoli cells were subjected to a brief hypotonic treatment using 20 mM Tris (pH 7.4) for 2.5 min to lyse residual germ cells [37]. These Sertoli cell cultures were ~98% pure, with negligible contamination of other testicular cells after Sertoli cell purity was assessed using specific Leydig, peritubular myoid, or germ cells markers by either immunoblotting (IB) or RT-PCR as described [38]. Sertoli cells cultured *in vitro* were shown to establish a functional TJ-permeability barrier with ultrastructures of TJs, basal ESs, gap junctions, and desmosomes that mimic the Sertoli cell BTB *in vivo* as described [39, 40]. The identity of Sertoli cells had been confirmed and characterized earlier using specific markers including electron microscopy [38, 39].

### Overexpression of F5-peptide in Sertoli cells

Laminin fragment F5-peptide was cloned into the pCI-neo mammalian expression vector (Promega, Madison, WI) as described [5]. All plasmids were prepared by Plasmid Plus Midi Kit (Qiagen, Chatsworth, CA) and confirmed by gene sequencing at Genewiz (South Plainfield, NJ). Sertoli cells were cultured *in vitro* for 3 days to allow assembly of a functional TJ-permeability barrier, containing ultrastructures of TJs, basal ESs, gap junctions and desmosomes when examined by electron microscopy [39, 40]. These cells were transfected with 0.25 μg (for IF on coverslips placed in 12-well plates), 1 μg (for RT-PCR and IB in 12-well plates) and 1.8 μg (for actin bundling assay in 6-well plates) plasmid DNA using Lipojet™ *in vitro* transfection reagent (SignaGen Laboratories, Rockville, MD) using a transfection medium: DNA ratio of 3:1. After 24 hr, cells were rinsed with F12/DMEM twice and then cultured in fresh F12/DMEM supplemented with growth factors and gentamicin. RNA and protein lysates were obtained from these cultures 48 and 72 hr post-transfection, respectively. For IF, Sertoli cells were terminated at 48 hr post-transfection. In selected experiments, plasmid DNA was labeled with Cy3 using Label/IT Tracker Intracellular Nucleic Acid Localization Kit (Mirus Bio, Madison, WI) to confirm successful transfection with a routine transfection efficiency of ~90%.

### Overexpression of F5-peptide in adult rat testes

To overexpress F5-peptide *in vivo*, adult male rats (~270–300 g b.w.) were transfected with pCI-neo (control, empty vector only) and pCI-neo/F5 (pCI-neo plasmid containing the F5-peptide clone) plasmid DNA via intratesticular injection using a 29-gauge needle as described [41]. The major difference between these methods, which used Polyplus *in vivo*-jetPEI^®^, and the one previously used by us is that the transfection efficiency was ~55% compare to *~*30% using conventional transfection medium [41]. One testis of each rat received pCI-neo vector alone, while the other testis received pCI-neo/F5 using Polyplus *in vivo*-jetPEI^®^ (Polyplus transfection S.A., Illkirch, France). In brief, plasmid DNA (15 μg) was constituted in 100 μl of transfection solution containing ~2.4 μl *in vivo*-jetPEI^®^ according to the manufacturer's instruction with an N/P ratio = 8 (note: N/P ratio is a measure of the ionic balance of the plasmid DNA, and it refers to the number of nitrogen residues of jetPEI/nucleotide phosphate, in which the jetPEI concentration is expressed in nitrogen residues molarity in which 1 μg of plasmid DNA contains 3 nmol of anionic phosphate). This transfection solution was administered to each testis (a testis weight of ~1.6 gm was taken to correspond to a volume of ~1.6 ml) using a 29-gauge needle attached to a 0.5 ml-syringe. The needle was gently inserted from the apical to near the basal end of each testis vertically, and as the needle was withdrawn apically, transfection solution was released gently and gradually from the syringe so that the entire testis was gradually filled with the transfection solution that spread the entire testis to avoid an acute rise in intratesticular hydrostatic pressure. For regimen 1, the first transfection was performed on day 0, followed by a second transfection on day 3. Rats (*n* = 7) were euthanized on day 5. For regimen 2, the first transfection was performed on day 0, followed by a second transfection on day 6. Rats were euthanized on day 8 (*n* = 5) and day 11 (*n* = 6). Because the phenotypes of these two regimens were identical, data from both regimens were combined for analysis as specified in Figure legends. For q-PCR, IB and IF, testes from rats (*n* = 9) were snap frozen immediately in liquid nitrogen, and stored at −80°C until used. Testes from rats (*n* = 9) were fixed in Bouin's fixative to obtain paraffin sections for histological analysis using hematoxylin and eosin (H&E) staining and IHC analysis.

### Assessing the efficacy of F5-peptide overexpression in the testis

The full-length DsRed2 cDNA encoding for the *Discosoma sp.* red fluorescent protein DsRed2 was cloned using a specific DsRed2 primer pair (Table S2) with pIRES2-DsRed2 plasmid DNA (Clontech, Mountain View, CA) served as the template for PCR. The DsRed2 cDNA was then ligated to the *Mlu* I and *Xba* I sites of pCI-neo mammalian expression vector. 15 μg pCI-neo vector or pCI-neo/DsRed2 plasmid DNA was transfected into the testis using Polyplus *in vivo*-jetPEI^®^ as described above (*n* = 3 rats per group on days 0 and 3). Rats were euthanized on day 5 as noted in Regimen 1. Seminiferous tubules (~ 100 tubules per testis with *n* = 3 rats) were randomly selected and those with > 10 fluorescent DsRed2 aggregates in the epithelium of a tubule cross-section were scored as successfully transfected tubules.

### Lysate preparation and immunoblotting (IB)

Protein lysates were extracted from primary Sertoli cells or testes using IP lysis buffer [50 mM Tris, 0.15 M NaCl, 1% NP-40, 2 mM EGTA, and 10% glycerol (v/v), pH 7.4 at 22°C] supplemented with 1 mM 4-(2-aminoethyl)benzene sulfonyl fluoride hydrochloride, 1 mM sodium orthovanadate, 0.05 mM bestatin, 0.05 mM sodium EDTA, 15 μM E64, 1 mM pepstatin, 4 mM sodium tartrate dehydrate, 5 mM NaF, and 3 mM β-glycerophosphate disodium salt. Protein concentration was determined using DC Protein Assay kits from Bio-Rad (Hercules, CA). Equal amount of protein lysate were resolved by SDS-PAGE and transferred to nitrocellulose membrane (Bio-Rad) for immunoblot analysis with the corresponding primary and secondary antibody (Table S1) as described [41]. Target proteins were visualized by enhanced chemiluminescence [42]. The images were acquired and quantified using a Fujifilm LAS-4000 mini-Luminescent Image Analyzer and Multi Gauge software package (Version 3.1) from Fujifilm Corp (Valhalla, NY).

### Immunohistochemistry (IHC)

IHC was performed using Bouin's-fixed, paraffin-embedded 5 μm-thick testis cross-sections. In brief, the cross-sections were deparaffinized, rehydrated, and then subjected to antigen retrieval. Sections were blocked with 10% normal goat serum, incubated with α-tubulin or EB1 antibody (Table S1) overnight at 4°C, followed by an incubation with biotinylated secondary antibody, and then streptavidin-horseradish peroxidase (Life Technologies). Positive immunoreactivity was visualized by using aminoethyl carbazole (AEC) with kits from Life Technologies, which appeared as reddish-brown precipitates in IHC. Negative controls using the same concentration of normal mouse IgG to substitute the primary antibody or the omission of secondary antibody were also included in our experiments.

### Dual-labeled immunofluorescence (IF) microscopy

Dual-labeled IF was performed using 7 μm-thick frozen cross-sections of testes or Sertoli cells cultured on coverslips at a density of 0.04 × 10^6^ cells/cm^2^. Sections and/or cells were fixed with 4% PFA or methanol, permeabilized with 0.1% Triton X-100 in PBS, and subsequently blocked in 10% normal goat serum or 1% BSA in PBS. Sections and/or cells were then incubated with primary antibodies (Table S1) at 4°C overnight, followed by Alexa Fluor 488 (green) or Alexa Fluor 555 (red)-conjugated secondary antibodies (Life Technologies) for 1 hr. To visualize F-actin, sections and/or cells were incubated with fluorescein isothiocyanate (FITC)-conjugated phalloidin (Life Technologies) at 1:50 dilution for 1 hr. The slides were mounted in Prolong Gold Antifade reagent with 4′, 6-diamidino-2-phenylindole (DAPI, Life Technologies) to visualize cell nuclei. Fluorescence and IHC images were examined and acquired using a Nikon Eclipse 90i Fluorescence Microscope system equipped with a Nikon Ds-Qi1Mc and a Nikon DS-Fi1 digital cameras, with the Nikon NIS Elements Imaging Software (Nikon Instruments, Inc). Image files were then analyzed using Photoshop in Adobe Creative Suite (Version 3.0; San Jose, CA) for image overlay to assess protein co-localization. All sections of testes or Sertoli cells within an experimental group were processed simultaneously in a single experimental session to eliminate intraexperimental variations and each experiment was repeated using at least 3 different rat testes or Sertoli cell preparations. Sertoli cells were also processed but incubated with normal IgG of the corresponding animal species by substituting the primary antibody or the omission of secondary antibody, which served as negative controls. Controls were included in each experimental session, which yielded no detectable staining, confirming the immunofluorescent staining for a specific target protein was specific.

### BTB integrity assay

The BTB integrity was assessed by using a membrane permeable biotinylation reagent, EZ-Link Sulfo-NHS-LC-Biotin (Thermo Fisher Scientific, Waltham, MA), using the procedure as earlier described [43]. In brief, adult male rats (*n* = 3, 270~300 g b.w.) received pCI-neo and pCI-neo/F5 on day 0 and 3 as described above and terminated on day 5. For positive control, rats received CdCl_2_ (3 mg/kg b.w., i.p.) and terminated on 5 days which is known to induce irreversible BTB disruption [44]. Rats received no treatment served as negative controls. Rats were anesthetized by ketamine HCl (60 mg/kg b.w. administered i.m.) with xylazine as an analgesic (10 mg/kg b.w. administered i.m.) (Sigma Aldrich, St. Louis, MO). Testes were exposed through a small incision in the scrotum, 100 μl of 10 mg/ml EZ-Link Sulfo-NHS-LC-Biotin, a membrane permeable biotinylation reagent with a Mr of 556.59, freshly diluted in PBS containing 1 mM CaCl_2_ was gently loaded under the tunica albuginea via a 29-gauge needle so that the biotinylation reagent could diffuse across a disrupted BTB if it was compromised following overexpression of the F5-peptide *vs.* controls. After 30 min, rats were euthanized by CO_2_ asphyxiation. Testes were removed immediately and snap-frozen in liquid nitrogen. Frozen 10 μm-thick cross-sections were fixed at room temperature in 4% PFA for 10 min, followed by Alexa Fluor 488-streptavidin (1:250) for 30 min. Slides were mounted with Prolong Gold Antifade reagent with DAPI (Life Technologies). To yield semi-quantitative data for the assessment of BTB integrity, the distance (D) traveled by the biotin (D_Biotin_) from the basement membrane in the seminiferous tubule *vs.* the radius of a tubule (D_Radius_) was recorded. Approximately 50 randomly selected tubules from each testis of *n* = 6 rats (including negative and positive control) were analyzed with a total of 300 tubules for treatment vs. control groups. For oblique sections of tubules, D_Radius_ was obtained using the average of the shortest and the longest distance from the basement membrane.

### Actin bundling assay

Actin bundling assay using lysates of Sertoli cells was performed as earlier described [45] using Actin Binding Protein Spin-Down Assay Biochem Kits from Cytoskeleton (Denver, CO). In brief, 10 μl of cell lysate from two wells of a 6-well plates *vs.* 10 μl Tris lysis buffer (served as a negative control) was added to the F-actin microfilaments to assess the ability to induce actin bundling. This mixture was centrifuged at 14,000 *g* at 24°C for 5 min to sediment bundled F-actin in the pellet, whereas the linear and unbundled actin microfilaments remain in the supernatant. Each pellet fraction was resuspended in 30 μl sterile water and used in its entirety for immunoblotting with an actin antibody; however, only 5 μl supernatant of each sample was used for analysis to prevent overloading the gel with protein. This experiment was repeated with 4 independent experiments using different cell preparations.

### RNA isolation, RT-PCR and q-PCR

Total RNA was isolated from testes and Sertoli cells using Trizol reagent (Life Technologies) and reverse transcribed with M-MLV reverse transcriptase (Promega). RT products were used as templates for PCR with a primer pair specific for F5-peptide and S16 (Table S2). Co-amplifications of F5-peptide and S16 were both in linear phase and PCR was performed as described [41], and authenticity of the PCR product was confirmed by direct nucleotide sequencing at Genewiz (South Plainfield, NJ). For q-PCR, the mRNA level of F5-peptide was analyzed by QuantStudio™ 12K Flex Real-Time PCR System (Thermo Fisher Scientific) with Power SYBR Green Master Mix (Thermo Fisher Scientific) according to the manufacturer's instructions (*n* = 9, each in triplicates). GAPDH was used as an internal control for normalization. The specificity of the fluorescent signal was verified by both melting curve analysis and gel electrophoresis. The F5-peptide expression level was determined using the 2^−ΔΔCT^ method.

### Image analysis

For *in vitro* experiments that assessed the effects of overexpression of F5-peptide on target protein distribution in Sertoli cells at the cell-cell interface, at least 200 cells from 3-5 independent experiments were randomly selected from each group and examined as earlier described [46]. In brief, fluorescence intensity of a target protein (e.g., CAR, ZO-1) at the cell-cell interface between adjacent Sertoli cells was quantified at two opposite ends of a cell and at least 50 cells from each experiment were randomly selected and quantified. To assess changes in protein distribution, the relative fluorescence intensity of a target protein (e.g., N-cadherin, ß-catenin) at the cell cortical zone was also measured at two opposite ends of a cell from at least 50 cells in an experiment. Fluorescence intensity of each target protein in the seminiferous epithelium of testes from at least 3 different rats following *in vivo* overexpression was quantified using ImageJ 1.45 software (NIH, Bethesda, MD; http://rsbweb.nih.gov/ij). To assess the effects of F5-peptide overexpression on the integrity of the BTB, approximately 50 tubules from 6 rats were randomly selected from each group, including the positive and negative controls, and examined.

### Assessment of seminiferous tubule abnormalities

Using frozen sections of testes and DAPI staining, the criteria to assess abnormality in tubules following overexpression of F5-peptide in testes were: (i) germ cell loss in which > 15 spermatids/spermatocytes were found within a tubule lumen, and (ii) gross changes in the seminiferous epithelium in which few germ cells were found within the epithelium. About 150 randomly selected tubules from each testis (*n* = 4 rats per group, totaling ~600 tubules per group) were randomly scored. However, due to the limited resolution using frozen sections, other apparently normal testes using frozen sections that had defects in spermatid and phagosome transport were further examined and demonstrated by histological analysis using paraffin sections following hematoxylin and eosin staining as described [41]. As such, defects in spermatid and phagosome transport were not scored and included in analysis using frozen sections.

### Statistical analysis

Two regimens were used in this study. For regimen 1 (*n* = 7 rats), testes from 4 rats were frozen in liquid nitrogen to obtain frozen sections for IF and IB analysis, and testes from 3 rats were fixed in Bouin's fixative to obtain paraffin sections for histological analysis. For regimen 2 (*n* = 11 rats), testes from 5 rats were frozen in liquid nitrogen and testes from 6 rats were for histological analysis. Because phenotypes using these two regimens were similar, data were pooled for analysis. For studies using Sertoli cell cultures, triplicate coverslips, or dishes, were used. Each data point/bar graph is the mean ± SD of *n* = 3 to 5 in the *in vitro* experiments or *n* = 4 to 9 rats for the *in vivo* experiments. Statistical analysis was performed using the GB-STAT software package (Version 7.0; Dynamic Microsystems, Silver Spring, MD). Statistical analysis was performed by two-way ANOVA followed by Dunnett's test. In selected experiments, a Student's *t*-test was used for paired comparisons.

## SUPPLEMENTARY MATERIALS TABLES




