# Supplementary material for: F5-peptide induces aspermatogenesis by disrupting organization of actin- and microtubule-based cytoskeletons in the testis
Source: Oncotarget. 2016 Sep 7;7(39):64203–20. doi: 10.18632/oncotarget.11887 (PMC5325436; doi:10.18632/oncotarget.11887)
Supplement: Supplementary file 1 [file oncotarget-07-64203-s001.pdf]

# F5-peptide induces aspermatogenesis by disrupting organization of actin- and microtubule-based cytoskeletons in the testis

## Supplementary Materials

**Supplementary Table S1: Antibodies used for different experiments in this study.**
see Supplementary_Table_S1

**Supplementary Table S2: Primers used for RT-PCR, qPCR and cloning of DsRed2**

| Gene | Primer Sequence | Position | Length (bp) | Tm (°C) | Cycle No. | GenBank accession number |
|---|---|---|---|---|---|---|
| F5 | Sense: 5′-CCTCCAATCTACCCAGCTC-3′ | 1790–1808 | 162 | 54 | 29 | NM_001107830 |
| | Antisense: 5′-GGCCACTGGTCCAGATGCT-3′ | 1933–1951 | | | | |
| GAPDH | Sense: 5′-GCTGGTCATCAACGGGAAAC-3′ | 192–211 | 112 | | | NM_017008.4 |
| | Antisense: 5′-GGTGAAGACGCCAGTAGAC-3′ | 285–303 | | | | |
| S16 | Sense: 5′-TCCGCTGCAGTCCGTTCAAGTCTT-3′ | 15–38 | 385 | | | XM_341815 |
| | Antisense: 5′-GCCAAACTTCTTGGTTTCGCAGCG-3′ | 376–399 | | | | |
| DsRed2 | Sense:5′-AA*ACGCGT*ATGGCCTCCTCCGAGAACGTCAT-3′ | 1–23 | 678 | | | https://www.addgene.org/ browse/sequence_vdb/3177/ |
| | Antisense:5′-AA*TCTAGA*CTACAGGAACAGGTGGTGGCGG-3′ | 657–678 | | | | |

[*] *ACGCGT*, restriction site for *MluI*; *TCTAGA*, restriction site for *XbaI*.
