# Supplementary material for: F5-peptide induces aspermatogenesis by disrupting organization of actin- and microtubule-based cytoskeletons in the testis
Source: Oncotarget. 2016 Sep 7;7(39):64203–20. doi: 10.18632/oncotarget.11887 (PMC5325436; doi:10.18632/oncotarget.11887)
Supplement: Supplementary file 2 [file oncotarget-07-64203-s002.pdf]

Table S1 Antibodies used for different experiments in this study

Antibody	Host species	Vendor	Catalog no.	Working dilution	
				IB	IF/IHC
Actin	Goat	Santa Cruz Biotechnology	sc-1616	1:300	
Akt	Rabbit	Cell Signaling Technology	9272	1:1000	
p-Akt1 S473	Rabbit	Cell Signaling Technology	4060	1:1000	
p-Akt2 S474	Rabbit	Cell Signaling Technology	8599	1:1000	
Arp3	Mouse	Sigma-Aldrich	A5979	1:3000	1:50
α-tubulin	Mouse	Abcam	ab7291		1:300/1:500
β-catenin	Rabbit	Invitrogen, Life Technologies	71-2700	1:250	1:100
CAR	Rabbit	Santa Cruz Biotechnology	sc-15405	1:200	1:50
Claudin-11	Rabbit	Invitrogen, Life Technologies	36-4500		1:100
Eps8	Mouse	BD Biosciences	610143	1:5000	1:50
EB1	Mouse	Santa Cruz Biotechnology	sc-374474		1:50
Formin 1	Mouse	Abcam	ab68058	1:500	1:50
JAM-A	Rabbit	Invitrogen, Life Technologies	36-1700	1:250	
Laminin γ3	Rabbit	Cheng Lab			1:300
N-cadherin	Mouse	Invitrogen, Life Technologies	33-3900	1:200	1:100
Nectin-3	Goat	Santa Cruz Biotechnology	sc-14806		1:25
Nectin-3	Rabbit	Santa Cruz Biotechnology	sc-28637	1:200	
Occludin	Rabbit	Invitrogen, Life Technologies	71-1500	1:250	
Palladin	Rabbit	Proteintech	10853-1-AP	1:1000	1:100
Plastin 3	Rabbit	Abcam	ab137585	1:500	
ZO-1	Rabbit	Invitrogen, Life Technologies	61-7300	1:250	1:100
ZO-1	Mouse	Invitrogen, Life Technologies	339111		1:100
Goat IgG-HRP	Bovine	Santa Cruz Biotechnology	sc-2350	1:3000	
Rabbit IgG-HRP	Bovine	Santa Cruz Biotechnology	sc-2370	1:3000	
Mouse IgG-HRP	Bovine	Santa Cruz Biotechnology	sc-2371	1:3000	
Rabbit IgG-Alexa Fluor 488	Goat	Thermo Fisher Scientific	A-11034		1:250
Rabbit IgG-Alexa Fluor 555	Goat	Thermo Fisher Scientific	A-21429		1:250
Mouse IgG-Alexa Fluor 555	Goat	Thermo Fisher Scientific	A-21424		1:250
Mouse IgG-Alexa Fluor 488	Goat	Thermo Fisher Scientific	A-11029		1:250
Goat IgG-Alexa Fluor-488	Donkey	Thermo Fisher Scientific	A-11055		1:250
Biotinylated Mouse IgG	Horse	Vector Laboratories	BA-2000		1:300

Abcam, Cambridge, MA; Cell Signaling Technology, Danvers, MA; Santa Cruz Biotechnology, Santa Cruz, CA; Sigma-Aldrich, St. Louis, MO; Invitrogen, Life Technologies, Carlsbad, CA; Proteintech, Chicago, IL; BD Biosciences, San Jose, CA; Millipore Corp., Billerica, MA.
